# Clinical Profile and Treatment Response of Patients with Ocular Inflammation due to Presumed Ocular Tuberculosis: A Retrospective Study

**DOI:** 10.4274/tjo.galenos.2019.05874

**Published:** 2019-09-03

**Authors:** Suma Elangovan, Senthamarai Govindarajan, Lakshmi Mayilvakanam, Nithya Gunasekaran

**Affiliations:** 1 Department of Ophthalmology, ESIC Medical College and PGIMSR, The TN. Dr. MGR Medical University, Chennai, India; 2 Consultant Ophthalmologist, Chennai, India (Past Affiliation: ESIC Medical College and PGIMSR, K.K. Nagar, Chennai); 3 Consultant Ophthalmologist, Puducherry, India (Past Affiliation: ESIC Medical College and PGIMSR, K.K. Nagar, Chennai)

**Keywords:** Presumed ocular tuberculosis, tuberculosis, ocular inflammation, extrapulmonary tuberculosis

## Abstract

**Objectives:**

Ocular tuberculosis is an extrapulmonary tuberculous infection and has varying manifestations which pose a huge challenge to diagnosis and treatment. The purpose of this study is to describe the various clinical manifestations of ocular inflammations due to tuberculosis and to assess the response to treatment following antituberculous therapy (ATT) and corticosteroids in these patients.

**Materials and Methods:**

We performed a retrospective analysis of 29 patients with presumed ocular tuberculosis who were started on ATT and completed follow-up of at least 6 months after ATT was initiated. The data collected were: age at presentation, sex, laterality, presence or absence of pulmonary/extrapulmonary tuberculosis, history of exposure to tuberculosis, site of ocular involvement and duration of illness, visual acuity at presentation and at 6-month follow-up, and response to treatment.

**Results:**

Most of the patients were of economically productive age, between 21-60 years. This most common presentation in our study population was unilateral nongranulomatous anterior uveitis. In spite of the delay between symptom onset and start of therapy, favorable response was noted in 79.3% of patients at completion of 6 months of ATT. The various reasons for the delay in start of therapy were also evaluated.

**Conclusion:**

In this case series, we presented the various ocular manifestations and the difficulties faced in the diagnosis of presumed ocular tuberculosis. Outcomes of ATT were favorable in most of our patients. Thus, the clinician should exercise a very high degree of suspicion and should not withhold a trial of ATT.

## Introduction

Tuberculosis is one of the leading infectious causes of morbidity and mortality worldwide, especially in the developing world. Data from the World Health Organization states that tuberculosis infects approximately one-third of the global population.^1,2^ In 2016, there were an estimated 10.4 million new tuberculous cases worldwide. Sixty-four percent of the global burden was contributed by seven countries: India, Indonesia, China, Philippines, Pakistan, Nigeria, and South Africa.^3^ Under-reporting and under-diagnosis have been cited as the major challenges to the treatment of tuberculous infection.

Ocular inflammation due to tuberculosis occurs either because of direct invasion by the tuberculosis bacilli or as a result of immunogenic reaction due to the extraocular infective foci. The prevalence of presumed ocular TB has been reported to vary widely depending upon the population studied and the diagnostic methods used, ranging between 1.4 and 18% in various studies.^4,5,6,7,8,9,10,11^

Patients can present with a wide variety of clinical manifestations in the external eye such as lid granulomas, conjunctival ulceration, hypertrophied excrescences, scleritis, keratitis, and phylectenulosis, to name a few. Intraocular signs of inflammation due to tuberculosis may also be varied, including uveitis, choroidal tubercles, choroiditis, retinal vasculitis, and optic nerve involvement.^12,13,14^ Thus, a high degree of suspicion is needed to diagnose and treat ocular tuberculosis.^15^

A definitive diagnosis is possible only when the tubercle bacilli can be visualized in or cultured from or its DNA amplified from the involved tissue. Because this is difficult to achieve, tuberculosis is often presumed, as suggested by Gupta et al.^16^ in 2007. In 2014, Gupta et al.^17^ proposed a newer classification of ocular tuberculosis with confirmed, possible, and probable categories in an effort to include ambiguous cases.

Recognition of the clinical signs of tuberculosis is important, as most of these patients will be treated with corticosteroids, which may flare up latent infection if missed. It will also help us to tailor investigations and promote better decision-making for initiating treatment in such cases.

The treatment of ocular tuberculosis usually consists of the four-drug regimen isoniazid, rifampicin, pyrazinamide, and ethambutol (HRZE) for 2 months followed by isoniazid and rifampicin (HR) to be continued up to 6-12 months. Concomitant use of corticosteroids by all routes (oral/topical/periocular) is needed in all ocular tuberculosis patients depending upon the clinical presentation. In 2003, the Centers for Disease Control and Prevention recommended prolonged therapy in cases of tuberculous infection at sites which respond slowly to therapy. Hence, patients with confirmed or presumed intraocular TB may require prolonged therapy.^18^ The recently updated guidelines published in 2016 recommend 6-9 months of therapy for extrapulmonary tuberculosis, though clear-cut recommendations for confirmed and presumed ocular tuberculosis have not been proposed.^19^

The purpose of this study was to describe the course and outcome of treatment and the various clinical manifestations of ocular inflammations observed in our center due to tuberculosis involving the anterior and posterior segments of the eye. Specifically, our objectives were to assess response to treatment following antituberculosis therapy (ATT) and corticosteroids in patients with ocular inflammation due to presumed ocular tuberculosis, and to analyze the various ocular manifestations and disease course in these patients.

## Materials and Methods

We performed a retrospective evaluation of patient data from the departmental records after obtaining ethical clearance from our institutional ethical committee. These patients presented to our ophthalmology outpatient clinic between June 2014 and May 2016.

We included patients with ocular inflammation who received ATT for presumed ocular tuberculosis and completed follow-up of at least 6 months after treatment was initiated.

All patients with ocular inflammation due to other causes (infectious and noninfectious) and those who did not come for follow-up were excluded from the study.

The criteria used in our center for diagnosis of presumed ocular tuberculosis were the presence of suggestive ocular findings in combination with

- Positive tuberculin sensitivity test (greater than 10 mm induration)

and/or

- Positive chest x-ray or computed tomography (CT)

and/or 

- evidence of confirmed extrapulmonary tuberculosis

- and exclusion of other possible entities.

Clinical signs of ocular inflammation which were considered suggestive of tuberculosis included granulomatous and nongranulomatous anterior uveitis, scleritis, keratoconjunctivitis, intermediate uveitis, retinal vasculitis, and optic neuritis. Since this was a retrospective study, no working protocol had been followed for all cases. Instead, individualized patient workup was done according to clinical findings.

From the records, the details of the complete ophthalmic examination with measurement of visual acuity, tonometry, slit-lamp examination, and dilated fundus exam were noted. Other modalities such as fundus fluorescein angiography, optical coherence tomography, perimetry, and B-scan ultrasound were used only as and when required. Blood analyses comprising complete blood count, erythrocyte sedimentation rate, blood glucose evaluation, and inflammatory workup including antinuclear antibody, C-reactive protein, HLA-B27, rheumatoid factor, TORCH (toxoplasmosis, rubella cytomegalovirus, herpes simplex, and HIV), and serum angiotensin-converting-enzyme to rule out other possible etiologies were performed when needed before initiating ATT. Immunological workup for tuberculosis such as Quantiferon TB gold assay and Mantoux were also done. Radiological studies like chest x-ray were done in all patients and chest CT was performed as required by a pulmonologist. A chest physician/internist evaluated the patients for signs of extrapulmonary and pulmonary tuberculosis.

The parameters included in our study were: age at presentation, sex, laterality, presence or absence of pulmonary/extrapulmonary tuberculosis, history of exposure to tuberculosis, site of ocular involvement and duration of illness, visual acuity at presentation and at 6-month follow-up, and response to treatment.

Response to treatment was assessed based on visual improvement and clinical response, time of onset of clinical improvement, and development of other complications. We defined a favorable outcome as vision improvement of at least 2 lines on Snellen chart and/or a decrease in clinical signs of inflammation (difference of more than 2 grades of inflammation as per Standardization of Uveitis Nomenclature Working Group and National Institutes of Health guidelines).^20,21,22,23^

## Results

We included 29 patients in our study, of which 69% were female (20 women, 9 men). The majority of patients belonged to the 41-60 year age group (62%, 18 patients) in comparison to 10 patients (34%) in the 21-40 year age group.

Eighty-three percent of the patients had unilateral involvement, of which right eye involvement was more common. The patients’ demographic characteristics are presented in Table 1. Four of the 29 patients were diabetics on oral antidiabetic therapy.

Thirty-eight percent (n=11) of the patients were found to have evidence of tuberculosis other than the ocular involvement. Five patients had active pulmonary tuberculosis and four showed evidence of old pulmonary tuberculosis or hilar adenopathy. One patient had cervical adenopathy with histopathological evidence of tuberculosis and one patient was on treatment for genitourinary tuberculosis.

The various clinical presentations are shown in Table 2. The mean time from onset of clinical symptoms to the start of ATT was 47 days (ranged from 11 to 72 days) in 25 patients, as 4 patients developed ocular symptoms while on ATT.

Chest x-ray and Mantoux test were performed for all patients. Among the 29 patients of this study, 90% showed strongly positive Mantoux test (<15 mm) and 10% showed Mantoux response of 10-15 mm. None of the patients had a Mantoux response of less than 10 mm.

Quantiferon TB gold assay was performed for 21 patients out of the 29, all of whom were found to be positive. Quantiferon test was found to be positive for the 3 patients who had Mantoux test results between 10-15 mm.

The main cause for delayed initiation of ATT was the time taken for the extensive laboratory and clinical workup. The various reasons for the delay in start of therapy are presented in Table 3.

Twenty-eight patients had been treated with category I ATT consisting of a 4-drug regimen (HRZE) for 2 months followed by a 2-drug regimen (HR) for 4 months. One patient was given category II therapy as he was a defaulter with active pulmonary tuberculosis. Concomitant oral/topical/periocular corticosteroid and cycloplegic therapy was administered depending upon the clinical presentation and need.

Seventeen patients on Category I completed 6 months of ATT. Twelve patients were subjected to 9 months of therapy. Five of these 12 patients had good resolution of symptoms after ATT and were given another 3 months of therapy. Four had relapses of inflammation and hence were treated with 9 months of ATT. Three patients had ocular inflammation with active pulmonary TB and were given therapy for up to 9 months.

Favorable clinical response was noted in 22 patients (75.9%) at 4-week follow-up after starting ATT and steroids. Two patients did not attend the 4-week follow-up but showed a favorable response when they visited at 7 and 8 weeks, respectively. Five patients showed minimal or no resolution of inflammation at 4 weeks. Their outcomes were as follows in subsequent follow-ups:

- Patient 1: presented with scleral inflammation, had recurrent attacks of scleritis and developed progressive scleral thinning and panuveitis through the ATT period, and developed staphyloma. Patient was re-evaluated after 2 months of ATT, but no other cause for the inflammation was ascertained.

- Patient 2: Presented with retinal periphlebitis (Eales disease), developed recurrent inflammation, vitreous hemorrhage and tractional retinal detachment, and underwent retinal detachment surgery.

- Patient 3: Developed recurrent bilateral corneal inflammation and pannus with corneal opacification in both eyes

- Patient 4: Developed recurrent intermediate uveitis and cystoid macular edema while on ATT

- Patient 5: Presented with choroiditis and post-uveitis, initially showed favorable improvement but relapsed in month 5 of ATT and was restarted on steroid therapy, developed retinal detachment.

We compared the patients’ best corrected visual acuity (BCVA) at presentation and at completion of 6 months of ATT. Six patients (20.6%) had worsening of presenting BCVA (caused by optic atrophy in 1 patient, staphyloma and retinal detachment in 1, corneal opacity in 1, macular scar in 1, and retinal detachment in 2 patients). Twenty-three patients (79.3%) maintained presenting BCVA or had improvement in BCVA.

Twenty-five of the 29 patients were followed up for an additional 6 months after completion of ATT (1 year of follow-up in total). Four patients, 2 with favorable and 2 with unfavorable outcomes at 6 months, were lost to follow-up after 6 months. Twenty-one of the 25 followed patients maintained their visual improvement (including 2 patients who underwent cataract surgery during the period) and were symptom-free for a year after starting ATT. One patient developed secondary glaucoma during this period of extended follow-up.

In total, 4 patients underwent uneventful cataract surgery for complicated cataract, 2 during the course of ATT and 2 after completing ATT. Two patients underwent surgery for retinal detachment during the course of ATT.

## Discussion

Extrapulmonary tuberculosis, especially ocular tuberculosis, poses many diagnostic and treatment dilemmas such as the lack of specific disease entities or clinical findings, protean manifestations, and multiple limitations to confirmatory diagnostic procedures. Therefore, most cases are diagnosed presumptively and not definitively after excluding other possible etiologies. Response to ATT may also provide indirect evidence for the correct diagnosis.

The patients who were analyzed were the economically productive strata between 21-60 years, with 62% belonging to age group of 41-60 years. Although tuberculosis is a systemic affliction, 83% of the study group showed unilateral involvement. The predominant clinical presentation in our group of patients was anterior uveitis (41% of patients) rather than posterior uveitis, which has been the commonest presentation in previous reports.^16,24^ In addition, the anterior uveitis was nongranulomatous in the majority of patients rather than the granulomatous variety which is more suggestive of tuberculosis.^16,24^

Tuberculosis elsewhere in the body was present in 38% and history of exposure to tuberculosis was elicited in only 20.7% of our patients. This shows that we cannot rely on history of exposure or presence of tuberculous lesions elsewhere as reliable criteria for diagnosis of ocular TB.

Ocular diagnostic methods like PCR assays require invasive tissue/sample procurement, are expensive and not readily available, and cannot be performed in all patients. We still have to rely on non-ocular methods like Mantoux test and gamma interferon release assays like Quantiferon Gold TB assay. When used judiciously, they have been shown to provide reliable results.^25^

In this study group, 90% of patients showed a strongly positive reaction to Mantoux test. Only 3 out of 29 showed reactions of 10-15 mm but they tested positive on Quantiferon TB assay. This clearly demonstrates that the Mantoux test cannot be disregarded as a way of evaluating a presumed ocular TB patient. It is also among the easiest tests to perform and is still largely available in many centers across India. A study conducted in Iraq showed high sensitivity and specificity of tuberculin sensitivity test with more than 14 mm induration.^26^ Many researchers recommend using combination of Mantoux test, interferon gamma release assays, and clinical signs to diagnose ocular tuberculosis.^27,28,29,30^ Ang et al.^31^ suggested that interferon assays should be preferred in areas where the prevalence of infection is low, and tuberculin skin test should be preferred where there is high prevalence of TB associated infection. Sudharshan et al.^32^ in their study established the utility of the Quantiferon TB Gold test in suspected TB uveitis.

We observed a mean duration of 47 days between symptom onset and start of therapy. The main reasons for the delay were the time required for the extensive workup to rule out other causes and hesitancy on the part of the clinician to start ATT without definitive evidence of the disease.

Among our patients, favorable response to therapy was noted in 75.9% of patients after 1 month of ATT. At completion of 6 months of ATT, favorable response was noted in 79.3% of patients. Relapses were noted in 2 patients during ATT therapy, which were controlled by restarting steroids. Five patients (17%) worsened in spite of therapy. A study by Basu et al.^33^ also observed progressive inflammation following ATT initiation for presumed ocular TB.

We followed up 25 patients (4 patients were lost to follow-up) for a further 6 months (1 year total). Of these, 21 maintained their visual outcome at end of 1 year (2 underwent cataract extraction) and had no relapses during the follow-up period.

Our data suggest that most patients benefitted from the therapy in the short term. A study in the UK showed that a minimum of 6 months of therapy provided good visual outcomes in the majority of patients.^34^ Sanghvi et al.^35^ recommended a full 6-month course of ATT although it may not cure the uveitis. Recently, Damato et al.^36^ also observed that most patients showed improvement even when start of treatment was delayed. In 2016, Lee et al.^37^ reported a 60-70% resolution of uveitis after a full course of ATT.

Definitive diagnostic methods like PCR with ocular samples are invasive and expensive, and the facilities are not widely available. Hence, the utility of tuberculin skin sensitivity testing and interferon gamma release assays is to be considered. Though there are wide variations in recommendations, they must be interpreted with caution.^38,39,40^

Therefore, we feel there should be no hesitancy to consider TB etiology in ocular inflammation or to consider a clinical trial of therapy in suspicious cases. Bansal et al.^41^ have suggested that treatment with ATT decreased the recurrence rate of uveitis by two-thirds when compared to treatment with anti-inflammatory drugs like steroids. Also, early administration of steroids without starting ATT has been shown to produce detrimental effects and may lead to poorer visual outcomes.^42^ Ang et al.^43^ in 2012 showed an 11-fold reduction in recurrence of uveitis when ATT was given for more than 9 months. In their case series published in 2016, Özdal et al.^44^ showed that uveitis did not recur in the majority of patients on ATT.

### Study Limitations

Our study has certain limitations. We included only patients with ocular inflammation with adequate follow-up while on ATT until completion of therapy. In addition, we did not investigate age-matched controls, which would have given more insight into the reliability of the immunological tests in this group of patients. Furthermore, since the number of patients in each clinical category was not large enough, we could not assess response to therapy in each clinical category.

## Conclusion

In this case series, we present the various ocular manifestations and the difficulties faced in the diagnosis and treatment of presumed ocular tuberculosis. Outcomes of ATT were favorable for most of our patients, even in those with delay in initiation of therapy. Thus, the clinician should excise a very high degree of suspicion and should not withhold a trial of ATT.

Uncertainties in the management of ocular tuberculosis must be addressed by creating protocols to be followed by both ophthalmologists and pulmonologists and infectious disease specialists.

Because of such inadequacies in diagnosis and treatment, ocular TB is probably grossly under-reported. Diagnostic guidelines and a protocol for investigation should be formulated and followed to ensure uniformity in treatment.

As we battle presumptive ocular TB, we should address the need for a multicenter study with long-term follow-up, which will help us to formulate better diagnostic and treatment guidelines for managing this public health issue.

## Figures and Tables

**Table 1:**
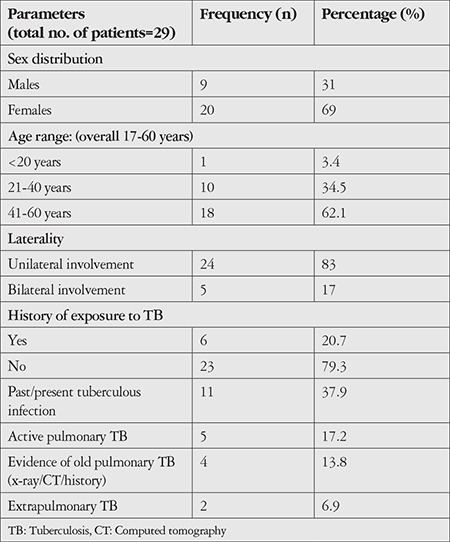
Characteristics of patients with presumed ocular tuberculosis

**Table 2:**
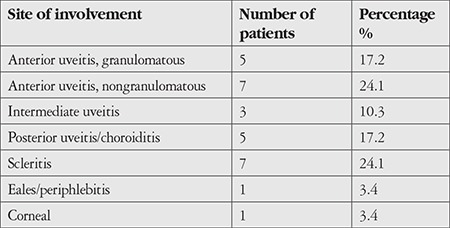
Clinical presentations of presumed ocular tuberculosis in our study population

**Table 3:**
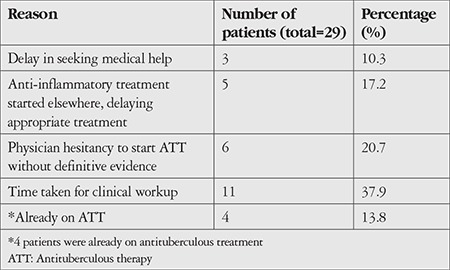
Reasons for delay between symptom onset and start of antituberculous therapy among patients with presumed ocular tuberculosis

## References

[1] Schlossberg D, Maher D, Raviglione MC (;Philadelphia; W.B Saunders Company). The global epidemic of tuberculosis: a World Health Organization perspective. In: Schlossberg D, ed. Tuberculosis and Nontuberculous Mycobacterial Infections (4th ed).

[2] Dye C, Scheele S, Dolin P, Pathania V, Raviglione MC (1999). Consensus statement. Global burden of tuberculosis: estimated incidence, prevalence, and mortality by country. WHO Global Surveillance and Monitoring Project. JAMA..

[3] GBD Tuberculosis Collaborators (2018). The global burden of tuberculosis: results from the Global Burden of Disease Study 2015. Lancet Infect Dis..

[4] Donahue HC (1967). Ophthalmologic experience in a tuberculosis sanatorium. Am J Ophthalmol..

[5] Wakabayashi T, Morimura Y, Miyamoto Y (2003). Changing patterns of intraocular inflammatory disease in Japan. Ocul Immunol Inflamm..

[6] Abrahams IW, Jiang YQ (1986). Ophthalmology in China. Endogenous uveitis in a Chinese ophthalmological clinic. Arch Ophthalmol..

[7] Islam SM, Tabbara KF (2002). Causes of uveitis at The Eye Center in Saudi Arabia: a retrospective review. Ophthalmic Epidemiol..

[8] Bouza E, Merino P, Munoz P, Sanchez-Carrillo C, Yanez J, Cortes C (1997). Ocular tuberculosis. A prospective study in a general hospital. Medicine (Baltimore)..

[9] Albert DM, Raven ML (2016). Ocular Tuberculosis. Microbiol Spectr..

[10] Rathinam SR, Cunningham ET Jr (2000). Infectious causes of uveitis in the developing world. Int Ophthalmol Clin..

[11] Abu El-Asrar AM, Abouammoh M, Al-Mezaine HS (2010). Tuberculous uveitis. Int Ophthalmol Clin..

[12] Gupta V, Shoughy SS, Mahajan S, Khairallah M, Rosenbaum JT, Curi A, Tabbara KF (2015). Clinics of Ocular Tuberculosis. Ocul Immunol Inflamm..

[13] Sharma A, Thapa B, Lavaju P (2011). Ocular tuberculosis: an update. Nepal J Ophthalmol..

[14] Alvarez GG, Roth VR, Hodge W (2009). Ocular tuberculosis: diagnostic and treatment challenges. Int J Infect Dis..

[15] Ang M, Chee SP (2017). Controversies in ocular tuberculosis. Br J Ophthalmol..

[16] Gupta V, Gupta A, Rao NA (2007). Intraocular tuberculosis- an update. Surv Ophthalmol..

[17] Gupta A, Sharma A, Bansal R, Sharma K (2015). Classification of intraocular tuberculosis. Ocul Immunol Inflamm..

[18] American Thoracic Society,, CDC,, Infectious Diseases Society of America (2003). Treatment of Tuberculosis. MMWR Recomm Rep..

[19] Nahid P, Dorman SE, Alipanah N, Barry PM, Brozek JL, Cattamanchi A, Chaisson LH, Chaisson RE, Daley CL, Grzemska M, Higashi JM, Ho CS, Hopewell PC, Keshavjee SA, Lienhardt C, Menzies R, Merrifield C, Narita M, O’Brien R, Peloquin CA, Raftery A, Saukkonen J, Schaaf HS, Sotgiu G, Starke JR, Migliori GB, Vernon A (2016). Executive Summary: Official American Thoracic Society/Centers for Disease Control and Prevention/Infectious Diseases Society of America Clinical Practice Guidelines: Treatment of Drug-Susceptible Tuberculosis. Clin Infect Dis..

[20] Jabs DA, Nussenblatt RB, Rosenbaum JT;, Standardization of Uveitis Nomenclature (SUN) working group (2005). Standardization of Uveitis Nomenclature for Reporting Clinical Data. Results of the First International Workshop. AM J Ophthalmol..

[21] Mahendradas P, Khanna A, Kawali A, Shetty R (2014). Quantification of inflammation in inflammatory eye diseases. IJRCI.

[22] Nussenblatt RB, Palestine AG, Chan CC, Roberge F (1985). Standardization of vitreal inflammatory activityin intermediate and posterior uveitis. Ophthalmology..

[23] McCluskey PJ, Wakefield D (1991). Prediction of response to treatment in patients with scleritis using a standardized scoring system. Aust N Z J Ophthalmol..

[24] Basu S, Monira S, Modi RR, Choudhury N, Mohan N, Padhi TR, Balne PK, Sharma S, Panigrahi SR (2014). Degree, duration, and causes of visual impairment in eyes affected with ocular tuberculosis. J Ophthalmic Inflamm Infect..

[25] Hong BK, Khanamiri HN, Bababeygy SR, Rao NA (2014). The utility of routine tuberculosis screening in county hospital patients with uveitis. Br J Ophthalmol.

[26] Al-Shakarchi FI (2014). Pattern of uveitis at a referral centre in Iraq. Middle East Afr J Ophthalmol..

[27] Babu K, Satish V, Satish S, SubbaKrishna DK, Abraham MP, Murthy KR (2009). Utility of QuantiFERON TB gold test in a south Indian patient population of ocular inflammation. Indian J Ophthalmol..

[28] Babu K, Philips M, Subbakrishna DK (2013). Perspectives of Quantiferon TB Gold test among Indian practitioners: a survey. J Ophthalmic Inflamm Infect..

[29] Ang M, Wong W, Ngan CC, Chee SP (2012). Interferon-gamma release assay as a diagnostic test for tuberculosis-associated uveitis. Eye..

[30] Ang M, Htoon HM, Chee SP (2009). Diagnosis of tuberculous uveitis: clinical application of an interferon-gamma release assay. Ophthalmology..

[31] Ang M, Wong WL, Li X, Chee SP (2013). Interferon γ release assay for the diagnosis of uveitis associated with tuberculosis: a Bayesian evaluation in the absence of a gold standard. Br J Ophthalmol..

[32] Sudharshan S, Ganesh SK, Balu G, Mahalakshmi B, Therese LK, Madhavan HN, Biswas J (2012). Utility of QuantiFERON(R)-TB Gold test in diagnosis and management of suspected tubercular uveitis in India. Int Ophthalmol..

[33] Basu S, Nayak S, Padhi TR, Das T (2013). Progressive ocular inflammation following anti-tubercular therapy for presumed ocular tuberculosis in a high-endemic setting. Eye (Lond)..

[34] Manousaridis K, Ong E, Stenton C, Gupta R, Browning AC, Pandit R (2013). Clinical presentation, treatment, and outcomes in presumed intraocular tuberculosis: experience from Newcastle upon Tyne, UK. Eye (Lond)..

[35] Sanghvi C, Bell C, Woodhead M, Hardy C, Jones N (2011). Presumed tuberculous uveitis: diagnosis, management, and outcome. Eye (Lond)..

[36] Damato EM, Dawson S, Liu X, Mukherjee C, Horsburgh J, Denniston AK, Moran E, Dedicoat M, Murray PI (2017). A retrospective cohort study of patients treated with anti-tuberculous therapy for presumed ocular tuberculosis. J Ophthalmic Inflamm Infect..

[37] Lee C, Agrawal R, Pavesio C (2016). Ocular Tuberculosis--A Clinical Conundrum. Ocul Immunol inflamm..

[38] Llorenç V, González-Martin J, Keller J, Rey A, Pelegrin L, Mesquida M, Adan A (2013). Indirect supportive evidence for diagnosis of tuberculosis-related uveitis: from the tuberculin skin test to the new interferon gamma release assays. Acta Ophthalmol..

[39] Denkinger CM, Dheda K, Pai M (2011). Guidelines on interferon-γ release assays for tuberculosis infection: concordance, discordance or confusion?. Clin Microbiol Infect.

[40] Kardos M, Kimball AB (2012). Time for a change? Updated guidelines using interferon gamma release assays for detection of latent tuberculosis infection in the office setting. J Am Acad Dermatol..

[41] Bansal R, Gupta A, Gupta V, Dogra MR, Bamberry P, Arora SK (2008). Role of Anti tubercular therapy in Uveitis with Latent/Manifest Tuberculosis. Am J Ophtalmol..

[42] Hamade IH, Tabbara KF (2010). Complications of presumed ocular tuberculosis. Acta Ophthalmol..

[43] Ang M, Hedayatfar A, Wong W, Chee SP (2012). Duration of anti-tubercular therapy in uveitis associated with latent tuberculosis: A case-control study. Br J Ophthalmol..

[44] Özdal PC, Tekin K, Özates S (2016). Current Approachesin Diagnosis and Treatment of Ocular Tuberculosis: A Case Series and Literature Review. Ret- Vit.

